# Parent–Adolescent Discrepancies in Perceived Parenting Characteristics and Adolescent Developmental Outcomes in Poor Chinese Families

**DOI:** 10.1007/s10826-013-9775-5

**Published:** 2013-05-29

**Authors:** Janet T. Y. Leung, Daniel T. L. Shek

**Affiliations:** Department of Applied Social Sciences, The Hong Kong Polytechnic University, Core H, Hung Hom, Hong Kong

**Keywords:** Poverty, Parenting, Parental control, Achievement motivation, Psychological competence, Adolescent

## Abstract

We examined the relationships between parent–adolescent discrepancies in perceived parenting characteristics (indexed by parental responsiveness, parental demandingness, and parental control) and adolescent developmental outcomes (indexed by achievement motivation and psychological competence) in poor families in Hong Kong. A sample of 275 intact families having at least one child aged 11–16 experiencing economic disadvantage were invited to participate in the study. Fathers and mothers completed the Parenting Style Scale and Chinese Parental Control Scale, and adolescents completed the Social-Oriented Achievement Motivation Scale and Chinese Positive Youth Development Scale in addition to paternal and maternal Parenting Style Scale and Chinese Parental Control Scale. Results indicated that parents and adolescents had different perceptions of parental responsiveness, parental demandingness, and paternal control, with adolescents generally perceived lower levels of parenting behaviors than did their parents. While father–adolescent discrepancy in perceived paternal responsiveness and mother–adolescent discrepancy in perceived maternal control negatively predicted adolescent achievement motivation, mother–adolescent discrepancy in perceptions of maternal responsiveness negatively predicted psychological competence in adolescents experiencing economic disadvantage. The present findings provided support that parent–child discrepancies in perceived parenting characteristics have negative impacts on the developmental outcomes of adolescents experiencing economic disadvantage. The present study addresses parent–child discrepancies in perceived parental behaviors as “legitimate” constructs, and explores their links with adolescent psychosocial development, which sheds light for researchers and clinical practitioners in helping the Chinese families experiencing economic disadvantage.

## Introduction

Parent–child discrepancies, like other informant discrepancies, have long been regarded as “‘methodological nuisances’ that needed to be ‘rectified’ in some way” (De Los Reyes 2011, p. 2). They were traditionally treated as measurement errors (McGuire 1969) instead of being legitimate constructs valuable for empirical study. Hence, with the exception of some isolated studies on parenting style (Paulson and Sputa 1996) and parenting behavior (De Los Reyes et al. 2010; Guion et al. 2009; Reynolds et al. 2011), very few studies have investigated the influence of parent–child discrepancies in perceptions of parenting behaviors on adolescent development. However, some researchers have argued that “informant discrepancies” are absolutely “more than measurement error” (Achenbach 2011, p. 80) because parent–child discrepancies have important meanings and implications for the clinical assessment of children and adolescents (Achenbach 2011; De Los Reyes 2011). As suggested by De Los Reyes (2011), informant discrepancies are important for understanding “the causes and consequences of, as well as treatments for, child and adolescent psychopathology” (p. 1), thus highlighting the legitimate nature of parent–adolescent discrepancies in perceptions of parenting in developmental research.

Theoretically, there are three accounts of parent–adolescent differences in their perceptions of parenting behaviors. First, developmental theorists interpret the differences as an indication of individuation (Grotevant and Cooper 1986), which is a normative developmental process. The discrepancies between adolescents and parents may be regarded as a manifestation of the adolescent desire for autonomy, independence and identity formation (Lerner and Spanier 1980). Second, parent–adolescent discrepancies may be interpreted in terms of the “generational stake” hypothesis. That is, parents have a stake in maximizing the similarities between themselves and their adolescent children, whereas adolescents have a stake in minimizing the similarities so as to display autonomy and independence (Bengtson and Kuypers 1971). Parents nurture their children, enhance family cohesion, and provide a healthy environment for the children. Thus, they have a tendency to portray their parenting behaviors as positive, as they have invested much time and effort in nurturing their children (Lerner and Knapp 1975; Lerner and Spanier 1980). On the other hand, adolescents focus on searching for self-identity and autonomy, thus enlarging the differences involved. The third explanation is operated in terms of parent–adolescent conflicts. Some family theorists suggest that parent–child discrepancies are the results of conflict between parents and adolescents. Olson et al. (1983) suggested that the stresses within the family result in different views of family processes among family members. Minuchin (1985) also suggested that parent–child discrepancies reflect family disorganization, maladaptive family interaction patterns, and a lack of cohesion. Under this perspective, different perceptions of family processes are associated with maladjustment of families, which in turn results in poor adjustment and negative psychological outcomes of adolescents (Guion et al. 2009; Welsh et al. 1998). According to the first two perspectives, discrepancies should not have a strong link to adolescent developmental outcomes. In contrast, the third perspective suggests that parent–child discrepancies in perceived parenting would be negatively related to child developmental outcomes.

There are several limitations on the literature on impact of parent–adolescent discrepancies in perceptions of parenting and adolescent developmental outcomes. First, the available findings are not conclusive. While there is evidence showing that higher parent–adolescent discrepancies in perceived parenting behaviors were related to healthier functioning of adolescents (Carlson et al. 1991; Holmbeck and O’Donnell 1991), there is also evidence indicating that higher parent–adolescent discrepancies were related to low levels of adolescent self-competence, self-esteem, social competence (Guion et al. 2009) and greater levels of adolescents’ internalizing and externalizing problems (De Los Reyes et al. 2010; Guion et al. 2009). Besides, fathers are usually ignored in the existing studies (De Los Reyes et al. 2010; Reynolds et al. 2011) and the sample size was small (e.g., Carlson et al. 1991; Fung and Lau 2010).

Second, there are few studies in which different measures of parenting are included in a single study. According to Darling and Steinberg (1993), two aspects of parenting (global parenting style and specific parenting behavior) were proposed. Maccoby and Martin (1983) classified parenting style in a two-dimensional framework: parental demandingness and parental responsiveness. Baumrind (1991) further elaborated: “demandingness refers to the claims parents make on the child to become integrated into the family whole by their maturity demands, supervision, disciplinary efforts and willingness to confront the child who disobeys. Responsiveness refers to actions which intentionally foster individuality, self-regulation and self-assertion by being attuned, supportive and acquiescent to the child’s special needs and demands” (p. 748). Parenting practices are “behaviours defined by specific context and socialization goals” (Darling and Steinberg 1993, p. 492). According to Shek (1999), few studies included these two aspects in a single study.

Third, as far as child developmental outcomes are concerned, most studies have focused on the relationship between parenting and adolescent developmental problems, such as internalizing and externalizing behaviors (e.g., Conger et al. 1994; Goosby 2007). Conceptually, psychological well-being can be defined in terms of the absence of manifested psychiatric symptoms or the presence of positive mental health attributes (Diener 1984) and coping strategies (Folkman et al. 1979). Unfortunately, positive mental health attributes of adolescents (e.g., purpose of life, hope, self-esteem) were relatively less focused in clinical family research.

Fourth, it was found that most related studies on parent–child discrepancies in perceived parenting behaviors were conducted in the Western world and there is little research data in Chinese contexts (Shek 2006). Studies in Chinese people are important because Chinese people constitute roughly one-fifth of the world’s population. Parent–child discrepancies may be intensified among Chinese families, where expectations about appropriate parenting behaviors may diverge as a function of differential orientations towards Chinese and Western cultural values (Fung and Lau 2010). For instance, Chinese parents may perceive punitive parenting as acceptable based on the Chinese belief of “*bang xia chu jiao zi*” (a filial son is the product of a rod). In contrast, under the influence of Western cultural beliefs on human rights and humanistic values, Chinese adolescents may perceive punitive parenting as an harm to parent–child relationship, or even as an abuse. They may adopt a lower level of acceptance of punitive parenting behaviors. With Westernization and urbanization, it is reasonable to expect there is the clash between traditional values (probably adopted by parents) and contemporary values (probably adopted by adolescents). Furthermore, there is the argument that as Chinese people grow up in a collectivistic culture rooted in Confucianism, Buddhism and Taoism whereas Western people grow up in an individualistic culture, family processes are different in the Chinese and non-Chinese contexts (Chao 1994). In the Chinese culture, parental control is a distinctive feature of Chinese parenting (Chao and Tseng 2002). As suggested by Chao (1994), parental control is associated with the concept of “training”, as expressed by the terms “*jiao xun*” (to train) and “*guan*” (to govern). Hence it would be enlightening to include parental control in examining parent–child discrepancies in perceived parenting.

Finally, the relationship between parent–adolescent discrepancies in perceived parenting and adolescent developmental outcomes is relatively unexplored in the context of poverty. There are two justifications why this should be examined in families experiencing economic disadvantage. First, there is a need to examine the generalizability of the related findings obtained in the general populations in different populations, such as the poor populations (i.e., generalizaility of findings). Second, because of limited financial resource, family social capital such as good parent–child relationship is important for poor families. As a legitimate dimension of family social capital, parent–child discrepancies in perceived parenting should be examined.

With reference to poor adolescents, two positive psychosocial attributes, including achievement motivation and psychological competence, are of interest to researchers. Achievement motivation is defined as “attention to a need of achievement” (Weiner 1992, p. 168). It is a “hope-oriented” attribute (Feather 1965) that measures one’s desire to achieve success based on the socially defined and approved standards emphasized in the Chinese culture (Yang and Yu 1988). For psychological competence, it is a composite of positive youth development attributes that include resilience, self-efficacy, sense of mastery, purpose of life and positive future orientation (Catalano et al. 2002; Shek et al. 2007). The study of achievement motivation and psychological competence is critical for adolescents experiencing economic disadvantage for three reasons. First, achievement motivation and psychological competence are important attributes of resilience (Benson 1997; Masten and Coatsworth 1998) which portray the capabilities of adolescents to bounce back in face of poverty and adversity. Second, both attributes are positive psychosocial measures of the assets, abilities, and potential of adolescents (Damon 2004; Shek et al. 2007), in contrast to the “deficiency” paradigm of looking into the problems and deficits. As suggested by Luthar et al. (1997) that “greater attention to theoretical conceptualizations regarding ‘normative development’, in the context of poverty” (p. 579), the employment of positive attributes allows us to have a holistic view on adolescent psychosocial capabilities. Last but not least, achievement motivation is a “hope-oriented” attribute (Feather 1965) that steers adolescents to succeed, which is especially important for economically disadvantaged adolescents to climb up the social ladder and escape from poverty.

### The Present Study

Against the above background, the study aimed to explore the relationships between parent–adolescent discrepancies in perceived parenting and adolescent positive development. We examined three research questions in this study:

1. Are there any differences in the perceptions of parenting characteristics (indexed by parental responsiveness, demandingness, and control) among fathers, mothers, and adolescents experiencing economic disadvantage? Based on the existing literature (Guion et al. 2009; Reynolds et al. 2011) and theoretical accounts of adolescent individuation process (Grotevant and Cooper 1986), and the “generational stake” thesis (Bengtson and Kuypers 1971), it was hypothesized that adolescents would have less positive perceptions of parenting than did their parents (Hypothesis 1).
2. Are there any relationships between parent–adolescent discrepancies in perceived parenting characteristics (indexed by parental responsiveness, demandingness, and control) and psychosocial development (indexed by achievement motivation and psychological competence) of economically disadvantaged adolescents? Based on the systems perspective that conflictual parent–child interactions may reflect family disorganization and lack of family cohesion (Minuchin 1985), it was hypothesized that greater discrepancies between fathers (and mothers) and adolescents in the perceptions of parenting would be associated with poor adolescent development (Hypothesis 2).
3. What is the relative importance of father–adolescent and mother–adolescent discrepancies in perceptions of parenting characteristics (indexed by parental responsiveness, demandingness, and control) in predicting achievement motivation and psychological competence of economically disadvantaged adolescents? This question is important for us to understand which parent–child discrepancies in parenting characteristics influence adolescent psychosocial development in poor families, and the results may shed light for clinical practitioners to formulate more effective strategies in family intervention. Furthermore, the findings will enable us to construct theoretical models on the relationship between parent–adolescent discrepancies in perceived family processes and adolescent psychosocial development. This is important because there is a severe lack of theoretical models in this area. In Chinese culture, fathers are obliged to take up the role of training and monitoring the behaviors of children, whereas mothers are caregivers, responsible for maintaining the childcare and household management (Shek 2002). Thus, fathers’ influence on adolescent achievement motivation and mothers’ influences on adolescent psychological competence may be substantial. As such, it was hypothesized that father–adolescent discrepancies in parental control would negatively influence adolescent achievement motivation (Hypothesis 3a), and mother–adolescent discrepancies in parental responsiveness would negatively influence adolescent psychological competence (Hypothesis 3b) in economically disadvantaged families.

## Method

### Participants

Intact Chinese families having at least one child aged 11–16 experiencing economic disadvantage were invited to participate in the study. In case a family had more than one child in the age group of 11–16, the elder child was invited to participate in the research as he/she had higher literacy level. The concept of relative poverty was adopted, with 50 % of monthly median domestic household income according to Hong Kong Population By-census 2006 used as the poverty threshold. There were 276 families participated in the study. There was one set of invalid questionnaires, leaving 275 sets of questionnaires for analyses.

### Measures

#### Parenting Characteristics

Paternal Parenting Style Scale (PPS/APPS) and Maternal Parenting Style Scale (MPS/AMPS) were used to measure the paternal and maternal demandingness and responsiveness. Based on the framework of Maccoby and Martin (1983) and parenting assessment work of Lamborn et al. (1991), Shek (1999) developed a modified version of the Paternal/Maternal Parenting Style Scale (PPS/MPS). There are two subscales: (1) Paternal/Maternal Demandingness Subscale (PDEM/MDEM) assessing demandingness of the father and mother towards the child’s behaviors; and (2) Paternal/Maternal Responsiveness Subscale (PRES/MRES) assessing responsiveness of the father and mother to the child’s behaviors. There are 7 items in the Demandingness Subscale and 13 items in the Responsiveness Subscale. The scales and subscales were found valid and reliable in the Chinese culture with internal consistency, test–retest reliability, and concurrent validity (Shek 1999, 2003). The total score of each subscale was used as an indicator of the level of parental demandingness and responsiveness, with a higher score indicating more positive parental attributes. Example of PDEM/MDEM item is “I keep pushing my child to do his/her best in whatever he/she does” and that of PRES/MRES item is “My child can count on me to help him/her out, if he/she has some kind of problem”. Reliability analyses showed that Paternal/Maternal Demandingness Subscale and Paternal/Maternal Responsiveness Subscale perceived by parents (PDEM/MDEM and PRES/MRES) and adolescents (APDEM/AMDEM and APRES/AMRES) had acceptable reliability in this study (*α* = .75 for PDEM, .65 for MDEM, .75 for APDEM, .72 for AMDEM, .70 for PRES, .61 for MRES, .82 for APRES and .80 for AMRES, respectively).

Chinese Paternal Control Scale (APCS) and Chinese Maternal Control Scale (AMCS) were used to measure paternal and maternal control respectively. Based on a review of the literature, Shek (2005, 2007b) developed a twelve-item Chinese Paternal/Maternal Control Scale to assess control based on indigenous Chinese cultural beliefs. Adolescents are requested to rate the degree of agreement with each item on a 4-point scale ranging from “Strongly agree” to “Strongly disagree”. Example of the item is “My father expects me to be mature (*sheng xing*)”. The APCS and AMCS showed internal consistency and divergent validity in previous studies (Shek 2007b). A higher total score of the measure indicates a higher level of Chinese parental control. The parental version of the scale (PCS/MCS) was modelled from adolescent’s version of Chinese Paternal/Maternal Control Scale respectively. Reliability analyses showed that PCS, MCS, APCS and AMCS had satisfactory reliability in this study (*α* = .85 for PCS, .87 for MCS, .87 for APCS, and .88 for AMCS, respectively).

#### Adolescent Psychosocial Development

Social Oriented Achievement Motivation Scale (SOAM) is a self-reported culture-specific measure of Chinese achievement tendencies developed by Yu and Yang (1989). It contains 30 items that measures four aspects of achievement motivation: achievement value, achievement goal, achievement related behaviors and outcome evaluation defined by significant others, groups and society (Yu 1996). Example of SOAM item is “In order not to disappoint my parents, I always try to do what they expect”. Participants wer**e** requested to rate the degree of agreement with each item on a 6-point Likert scale ranging from “Strongly agree” to “Strongly disagree”. The scale has good internal consistency, test–retest reliability, convergent validity and discriminant validity (Yu and Yang 1989). The total score of the items in the scale is an indicator of the degree of social-oriented achievement motivation, with higher scores indicating higher levels. The scale was demonstrated to have good reliability in this study (*α* = .94).

Chinese Positive Youth Development Scale (CPYDS) is a 90-item Chinese psychosocial measure developed by Shek et al. (2007) in measuring positive youth development. As far as resilience and psychological competence of adolescents were concerned, seven positive youth development constructs measuring the psychological competence of adolescents (resilience, cognitive competence, self-determination, self-efficacy, spirituality, beliefs in the future, and clear and positive identity) were used in this study. Example of CPYDS item is “When I face difficulty, I will not give up easily”.The CPYDS showed acceptable internal consistency, criterion-related validity, construct validity and convergent validity in previous study (Shek et al. 2007). The scale was demonstrated to have excellent reliability in this study (*α* = .94).

### Procedures

We conducted a cross-sectional survey with purposeful sampling. Families experiencing economic disadvantage were identified and recruited by social workers of various children and youth service centers, school social work services, community centers and family service centers across Hong Kong. Training and briefing sessions were organized to participating social workers by the researchers on the identification of the respondent families as well as implementation of data collection. There were totally 10 non-governmental organizations and 24 social service units involved in the study. The data collection was either arranged in the social service units or at the respondents’ homes, according to the desire of the families. During data collection, fathers, mothers and adolescents were given explanations about the purpose of the research, procedure of data collection, the rights of respondents to voluntarily participate and withdrawal from the study, as well as the use of the data in the study. Written informed consent was obtained from all participants. Fathers and mothers were requested to complete the Father Questionnaire and the Mother Questionnaire respectively which contained identical measures of parental responsiveness, demandingness, and control, whereas adolescents were requested to complete the Adolescent Questionnaire which contained measures of paternal and maternal responsiveness, paternal and maternal demandingness, paternal and maternal control, achievement motivation, and psychological competence. To ensure confidentiality, the questionnaire was completed by each participant separately. The questionnaire was administered in a self-administered format. In case the participants had difficulties comprehending the questionnaires, the questions or items were read out by researchers or trained social workers in an interview format. Parents took around 45 min to 1 h to complete the questionnaires, depending on their literacy level. Adolescents took around 35 min to complete the questionnaires.

### Data Analysis Plan

To address Research Question 1, we performed multivariate analyses of variance (MANOVA), univariate analyses of variance (ANOVAs) and paired *t* tests to examine both father–adolescent and mother–adolescent differences in perceived parenting characteristics.

For Research Questions 2 and 3, discrepancy scores between parents and adolescents in perceived parenting characteristics were determined. In the present study, differences between the standardized ratings of fathers and adolescents, and between those of mothers and adolescents, were employed for four reasons. First, the standardized discrepancy scores allow the parents’ report and children’s reports to contribute equally to discrepancy scores of the variables. As explained by De Los Reyes and Kazdin (2004) that ‘no one informant can be considered a “gold standard” by which to interpret another informant’s ratings’ (p. 334), it is important that the calculated discrepancy scores correlate equally with the parents’ and children’s ratings so as to produce the most consistent estimates among informant discrepancies and informant characteristics (De Los Reyes and Kazdin 2004). In contrast, the discrepancy scores computed from raw data are affected by the differential distributions of the individual scores (Guion et al. 2009). Second, the standardized approach helps to adjust the systematic biases in variability of informant responses as it empirically equates the distributions of parents’ and adolescent’s ratings by the z distribution (Guion et al. 2009). This is important because adolescent’s ratings on family processes always had greater variability than parents’ ratings, resulting in higher correlation with the discrepancy scores when raw data were calculated. Third, the standardized approach enhances the interpretability of the score, with the standardized score has a mean of 0 and a standard deviation of 1. Fourth, as many studies of calculated parent–child discrepancy scores employ the standardized approach (De Los Reyes and Kazdin 2004; Guion et al. 2009), this maximizes comparability with related research on parent–child discrepancy and adolescent development.

When employing the standardized approach, the parents’ and adolescents’ reports on parenting characteristics were converted into z scores (the standardized scores). The discrepancy scores were calculated by subtracting the adolescents’ standardized scores from the parents’ standardized scores on various parenting characteristics. The positive discrepancy score shows that parents’ report was more positive than the adolescents’ report.

To address Research Question 2, Pearson correlation analyses were performed to analyse the relationships between parent–adolescent discrepancies in perceptions of various parenting characteristics and adolescent psychosocial development. A two-tailed multistage Bonferroni procedure was carried out to guard against inflated Type I error (Larzelere and Mulaik 1977).

For Research Question 3, standard multiple regression was performed to understand the overall influences of parent–child discrepancies in perceived parenting characteristics on achievement motivation and psychological competence of adolescents respectively.

## Results

### Descriptive Statistics

The mean ages of the fathers and mothers were 49.94 (SD = 9.28) and 42.18 (SD = 4.97), respectively. A majority of parents received less education, with 205 fathers (74.5 %) and 204 mothers (74.2 %) had junior secondary or lower educational level. There were 211 (76.7 %) fathers who had a job, whereas a high proportion of mothers were housewives (n = 199, 72.4 %). The average number of children in the families was 2.34 (SD = .90). There were 96 families receiving Comprehensive Social Security Assistance from the Government, representing 34.8 % of the sample.

For the adolescent sample, there were 134 boys (48.7 %) and 141 girls (51.3 %) participated in the study. The mean age of the adolescents was 13.56 (SD = 1.54), with the means of boys and girls at 13.40 (SD = 1.60) and 13.71 (SD = 1.47) respectively. There were 65 adolescents (23.6 %) studying in primary school (Grade 6 or below), 151 (54.9 %) in junior secondary level (Grade 7 to Grade 9), 57 (20.7 %) in senior secondary level (Grade 9 and above).

Descriptive statistics of the measures were shown in Table 1.

**Table 1:** Descriptive statistics of the measures

	Range	*M*	SD
*Parental demandingness*			
PDEM	2–16	11.71	3.36
MDEM	2–17	13.10	2.61
APDEM	0–16	9.51	4.03
AMDEM	0–16	11.41	3.49
*Parental responsiveness*			
PRES	2–21	15.47	3.28
MRES	8–22	17.04	2.77
APRES	0–22	12.53	4.50
AMRES	0–22	15.52	4.05
*Parental control*			
PCS	25–48	38.12	4.46
MCS	18–48	38.93	4.88
APCS	12–48	36.44	5.86
AMCS	13–48	38.42	5.78
Social oriented achievement motivation	35–180	117.93	25.08
Positive youth development	38–123	91.43	15.20

*Parental responsiveness* PRES = Paternal Responsiveness Subscale reported by fathers. APRES = Paternal Responsiveness Subscale reported by adolescents. MRES = Maternal Responsiveness Subscale reported by mothers. AMRES = Maternal Responsiveness Subscale reported by adolescents. *Parental demandingness* PDEM = Paternal Demandingness Subscale reported by fathers. APDEM = Paternal Demandingness Subscale reported by adolescents. MDEM = Maternal Demandingness Subscale reported by mothers. AMDEM = Maternal Demandingness Subscale reported by adolescents. *Parental control* PCS = Chinese Paternal Control Scale reported by fathers. APCS = Chinese Paternal Control Scale reported by adolescents. MCS = Chinese Maternal Control Scale reported by mothers. AMCS = Chinese Maternal Control Scale reported by adolescents

### Research Question 1

From the data of dyadic family processes of parental responsiveness, demandingness, and control, using Wilks’ criterion, the results of MANOVA indicated a significant overall main effect for the reporters (fathers’ reports, mothers’ reports, adolescents’ reports of perceived paternal and maternal dyadic parenting characteristics), with *F*(3,1096) = 39.19, *p* < .001, partial eta squared = .57. We performed univariate ANOVA in order to examine the differences in the individual dependent variable. Bonferroni correction (alpha = .05/4, i.e., .013) was adopted to reduce the chance of committing inflated Type I error. Significant effects were further analysed by post hoc comparisons of Tukey’s HSD calculation.

Univariate analyses of variance showed significant effect on parental responsiveness for different reporters, with *F*(3,1096) = 106.75, *p* < .001, partial eta squared = .28. With post hoc comparisons of Tukey’s HSD value, we found that there was significant difference on paternal responsiveness between fathers and adolescents, with adolescents perceiving lower level than did fathers. Similar findings occurred in mother–adolescent differences, with adolescents perceiving significantly lower level of maternal responsiveness than did mothers.

For parental demandingness, univariate ANVOA showed significant effect for different reporters, with *F*(3,1096) = 89.59, *p* < .001, partial eta squared = .25. With post hoc comparisons of Tukey’s HSD value, we found that there was significant father–adolescent difference on perceived demandingness, with adolescents perceiving lower level than did fathers. Similar findings occurred in mother–adolescent differences, with adolescents perceiving significantly lower level of maternal demandingness than did mothers.

For parental control, univariate ANVOA showed significant effect for different reporters, with *F*(3,1096) = 15.68, *p* < .001, partial eta squared = .05. We found that there was significant difference on paternal control between fathers and adolescents, with adolescents perceiving lower paternal control than did fathers. However, there was no significant difference on maternal control between mothers and adolescents. In summary, Hypothesis 1 was generally supported (see Table 2).

**Table 2:** Effects and post hoc comparison of different informants on the measures of parenting characteristics

	Effect		Post-hoc comparison			
	*F* value	*Partial η²*	A (fathers vs. mothers)	B (paternal vs. maternal processes by adolescents)	C (fathers vs. paternal processes by adolescents)	D (mothers vs. maternal processes by adolescents)
*Measure*						
Parental responsiveness	106.75***	.28	S (M > F)	S (A_m_ > A_p_)	S (F > A_p_)	S (M > A_m_)
Parental demandingness	89.59***	.25	S (M > F)	S (A_m_ > A_p_)	S (F > A_p_)	S (M > A_m_)
Parental control	15.68***	.05	NS	S (A_m_ > A_p_)	S (F > A_p_)	NS

Post-hoc comparisons: *A* father–mother difference of the measure. *B* Paternal and maternal difference of the measure by the adolescents. *C* father–adolescent difference of the measure. *D* mother–adolescent difference of the measure. S significant at .05 % level. *M* > *F* Mothers’ scores higher than fathers’ scores. *A*_*m*_ > *A*_*p*_ Adolescents’ perceived maternal scores higher than perceived paternal scores. *F* > *A*_*p*_ Fathers’ scores higher than adolescents’ perceived paternal scores. *M* > *A*_*m*_ Mothers’ scores higher than adolescents’ perceived maternal scores

*NS* not significant

* *p* < .05; ** *p* < .01; *** *p* < .001

To assess the effect size of dyadic discrepancies in parental responsiveness, demandingness, and control, partial eta squared was calculated. It was found that partial eta squared of father–adolescent and mother–adolescent discrepancies in perceived responsiveness and demandingness were .29 and .13 respectively, which were considered as great according to Stevens’s (2002) suggestion (.01 = small effect, .06 = medium effect, .14 = large effect). Besides, mother–adolescent discrepancies in maternal responsiveness and demandingness were also great, with partial eta squared of .22 and .19 respectively. When comparing the magnitude of the effect size, we found that father–adolescent discrepancies in parental responsiveness and control were greater than mother–adolescent discrepancies, whereas mother–adolescent discrepancies in parental demandingness had greater effect size than father–adolescent discrepancies (see Table 3).

**Table 3:** Effect size (partial eta squared) of dyadic discrepancies on different parenting characteristics

	Father–adolescent discrepancy	Mother–adolescent discrepancy
	Effect size (*Partial η²*)	Effect size (*Partial η²*)
Parental responsiveness	.29	.22
Parental demandingness	.13	.19
Parental control	.06	.01

To address the issue of non-independence of observations in MANOVA, separate paired *t* tests were performed for each measure with Bonferroni correction (alpha = .05/6, i.e., .008). Findings similarly showed that there was significant difference between parents’ and adolescents’ perceptions of perceived demandingness and responsiveness, and between fathers’ and adolescents’ perceptions of perceived control. The findings can be seen in Table 4.

**Table 4:** Mean differences on perceptions of parenting characteristics between parents and adolescents

Variables of family processes	Variables in comparison	*df*	*t* values	Effect size (Cohen’s *d*)
Parental responsiveness	Fathers’ perceptions (PRES) versus adolescents’ perceptions (APRES)	274	10.69***	.76
	Mothers’ perceptions (MRES) versus adolescents’ perceptions (AMRES)	274	6.42***	.45
Parenting demandingness	Fathers’ perceptions (PDEM) versus adolescents’ perceptions (APDEM)	274	8.85***	.59
	Mothers’ perceptions (MDEM) versus adolescents’ perceptions (AMDEM)	274	8.03***	.55
Paternal control	Fathers’ perceptions (PCS) versus adolescents’ perceptions (APCS)	274	4.25***	.32
	Mothers’ perceptions (MCS) versus adolescents’ perceptions (AMCS)	274	1.29 NS	.10

PRES = Paternal Responsiveness Scale completed by fathers. MRES = Maternal Responsiveness Scale completed by mothers. APRES = Paternal Responsiveness Scale completed by adolescents. AMRES = Maternal Responsiveness Scale completed by adolescents. PDEM = Paternal Demandingness Scale completed by fathers. MDEM = Maternal Demandingness Scale completed by mothers. APDEM = Paternal Demandingness Scale completed by adolescents. AMDEM = Maternal Demandingness Scale completed by adolescents. PCS = Chinese Paternal Control Scale completed by fathers. MCS = Chinese Maternal Control Scale completed by mothers. APCS = Chinese Paternal Control Scale completed by adolescents. AMCS = Chinese Maternal Control Scale completed by adolescents

*NS* not significant

* *p* < .008 (Bonferroni correction was adopted to guard against familywise Type I error); *** *p* < .001

### Research Question 2

Correlation analyses suggested that there were no significant relationships between demographic data (including adolescent age, gender, educational level, duration of stay in Hong Kong), parent–child discrepancies in perceived parenting characteristics and adolescent development.

We found that father–child discrepancies in perceived paternal responsiveness and demandingness, and mother–child discrepancy in perceived maternal control were associated negatively with achievement motivation of economically disadvantaged adolescents, with Pearson’s r of −.18 (*p* < .006, two-tailed multistage Bonferroni procedure was carried out), −.12 (*p* < .05, marginally significant), −.18 (*p* < .006), respectively, indicating small to medium effect size. Besides, it was found that father–child and mother–child discrepancies in perceived parental responsiveness were negatively correlated with psychological competence of adolescents, with Pearson’s r of −.15 (*p* < .05 marginally significant) and −.18 (*p* < .006), respectively. Also, father–child and mother–child discrepancies in perceived parental control were found negatively correlated with psychological competence of adolescents, with Pearson’s r of −.18 (*p* < .006) and −.17 (*p* < .006), respectively. Hypothesis 2 was partially supported (see Table 5).

**Table 5:** Correlations between parent–child discrepancies in perceived parenting characteristics and adolescent psychosocial development in economically disadvantaged families

	Parent–child discrepancies on parental responsiveness		Parent–child discrepancies on parental demandingness		Parent–child discrepancies on parental control	
	DIPRES	DIMRES	DIPDEM	DIMDEM	DIPCS	DIMCS
*Adolescents*						
SOAM	−.18*	−.11 NS	−.12^a^	−.00 NS	−.09 NS	−.18*
PYD	−.15^a^	−.18*	−.10 NS	−.05 NS	−.18*	−.17*

Bonferroni correction was used to evaluate the significance of the correlations

DIPRES = Discrepancy score on paternal responsiveness between fathers and adolescents. DIMRES = Discrepancy score on maternal responsiveness between mothers and adolescents. DIPDEM = Discrepancy score on paternal demandingness between fathers and adolescents. DIMDEM = Discrepancy score on maternal demandingness between mothers and adolescents. DIPCS = Discrepancy score on paternal control between fathers and adolescents. DIMCS = Discrepancy score on maternal control between mothers and adolescents. SOAM = Social Oriented Achievement Motivation Scale. PYD = Chinese Positive Youth Development Scale (with 7 subscales selected)

*NS* not significant

* Indicates that the r value is significant when familywise Type I error. *pFW* < .05, *pT* < .006

^a^Borderline correlation *p* < .05; *** *p* < .001

### Research Question 3

As only father–child discrepancies in perceived paternal responsiveness and demandingness, and mother–child discrepancy in perceived maternal control were significantly associated with adolescent achievement motivation, these three variables were considered as the predictor variables. We found that parent–child discrepancies in perceived parenting characteristics predicted the achievement motivation of economically disadvantaged adolescents (Multiple *R* = .24, *p* < .001), adding 6 % to the explained variance. Father–child discrepancy in paternal responsiveness and mother–child discrepancy in perceived maternal control were found negatively predicted adolescent achievement motivation, with *β* = − .15 (*p* < .05) and −.16 (*p* < .01) respectively. The effect size of Cohen *f* ^2^ was .06, which was considered between small and medium effect according to Cohen’s (1988) suggestion (.02 = small effect, .15 = medium effect, .35 = large effect).

As father–child and mother–child discrepancies in perceived parental responsiveness and control were associated negatively with adolescent psychological competence, these four variables were regarded as the predictor variables. We found that parent–child discrepancies in perceived parenting characteristics significantly predicted the psychological competence of economically disadvantaged adolescents (Multiple *R* = .28, *p* < .001), adding 8 % to the explained variance. Mother–child discrepancy in perceived maternal responsiveness was found negatively predicted adolescent psychological competence, with *β* = −.15 (*p* < .05) (see Table 6). The effect size of Cohen *f* ^2^ was .08, which was considered between small and medium effect according to Cohen’s (1988) suggestion.

**Table 6:** Prediction of parent–child discrepancies on perceived parenting characteristics on positive developmental outcomes of adolescents experiencing economic disadvantage

	Multiple R	Standardized regression coefficient (*β*)						*R* ^*2*^	Adjusted *R* ^2^	Cohen *f* ^2^
		Parent–child discrepancy on parental responsiveness		Parent–child discrepancy on parenting style		Parent–child discrepancy on parental control				
		DIPRES	DIMRES	DIPDEM	DIMDEM	DIPCS	DIMCS			
*Adolescents*										
SOAM	.24***	−.15*	NA	−.03 NS	NA	NA	−.16**	.06	.05	.06
PYD	.28***	−.05 NS	−.15*	NA	NA	−.13 NS	−.11 NS	.08	.06	.08

DIPRES = Discrepancy score on paternal responsiveness between fathers and adolescents. DIMRES = Discrepancy score on maternal responsiveness between mothers and adolescents. DIPDEM = Discrepancy score on paternal demandingness between fathers and adolescents. DIMDEM = Discrepancy score on maternal demandingness between mothers and adolescents. DIPCS = Discrepancy score on paternal control between fathers and adolescents. DIMCS = Discrepancy score on maternal control between mothers and adolescents. SOAM = Social Oriented Achievement Motivation Scale. PYD = Chinese Positive Youth Development Scale (with 7 subscales selected)

*NA* not applicable, *NS* not significant

* *p* < .05; ** *p* < .01; *** *p* < .001

## Discussion

This paper attempts to study the relationship between parent–adolescent discrepancies in perceptions of parenting characteristics and adolescent psychosocial development in Chinese families experiencing economic disadvantage. There are several unique features in the study. First, parent–adolescent discrepancy in perceived parenting characteristics was regarded as a “legitimate” measurement construct and its relationship with adolescent psychosocial development was explored. Second, members of Chinese families experiencing economic disadvantage were recruited as participants of the study, with both cultural and socio-economic contexts were unique in the related studies. Third, different parenting characteristics, including parenting styles (parental responsiveness and demandingness) and indigenously defined parenting practices (parental control), were analysed in the study. Fourth, achievement motivation and psychological competence, which are positive youth developmental attributes that emphasize adolescents’ capabilities in face of poverty and adversity, were employed as outcome measures in this study. Fifth, indigenous Chinese conceptions of parental control and achievement motivation were adopted. Last but not least, fathers, mothers and adolescents were recruited in the study.

The findings support the previous research (e.g., Paulson and Sputa 1996) that parents and adolescents had different perceptions of parenting behaviors, with adolescents showing less positive perceptions of parenting behaviors than did their parents. The present findings support the observations of Paulson and Sputa (1996) that “what parents think they may be doing in the home may not be what the adolescent perceives” (p. 371). Furthermore, echoing the literature that parent–child discrepancies in perceived family processes were related to low levels of adolescent psychosocial development (Guion et al. 2009; De Los Reyes et al. 2010), the findings revealed that higher parent–adolescent discrepancies in parenting characteristics was generally related to lower achievement motivation and psychological competence in economically disadvantaged adolescents, though the effects were between small to medium according to Cohen’s (1988) suggestion. The small effect may imply a weak relationship between parent–child discrepancies of perceived parenting characteristics and adolescent psychosocial development in poor families, it may also be due to the methodological limitation of purposeful sampling. Poverty may bring social stigmatisation that prohibited poor families to participate in the research, especially in the Chinese community where poverty is generally perceived as “losing face” and disgracing the family name. It was also found that poor families having better parenting qualities tend to participate more in family-related research than are those with poorer parenting qualities (Hoff et al. 2002). Thus, with relatively fewer families of poor parenting practice and negative adolescent behavioral outcomes having involved in the research, the effect of parent–child discrepancies of parenting characteristics in influencing adolescent psychosocial development may also be affected.

Nevertheless, the findings sound an alarm that parent–adolescent discrepancies in parenting behaviors may go beyond the normal and healthy developmental assumptions of the individuation process (Grotevant and Cooper 1986) and the “generational stake” thesis (Bengtson and Kuypers 1971). The directions of correlation coefficients suggest that there may be conflicting interactions between parents and adolescents in poor families, which may result in poor psychological adjustment of adolescents. This interpretation is in line with the clinical literature that discrepancies between family members in perceived parenting reflect a lack of harmony and cohesion in the family (Minuchin 1985).

It is noteworthy that father–adolescent discrepancies in perceived parental responsiveness appear to be greater that mother–adolescent discrepancies, whereas father–adolescent discrepancies in perceived parental demandingness were smaller than mother–adolescent discrepancies. According to the sex-role theory (Bem 1974), femininity is associated with expressiveness, whereas masculinity is associated with instrumentality (Spence 1993). Mothers may adopt a more affective style in family roles, whereas fathers may adopt a more goal-oriented style (Russell et al. 1998). Hence, fathers may seldom express their care and concern to their children directly, but will lay out goals and provide goal-oriented support to adolescents. Besides, based on the role theory of cultural perspectives, fathers are responsible for mobilizing financial resources for child development, whereas as mothers are responsible for taking care of their children. Thus, mothers are more involved in parenting than are fathers in the Chinese culture, as indicated by the Chinese cultural inclination of “*Nan zhu wai, nu zhu nei*” (men manage things outside the family; women manage things inside). This is more salient in economically disadvantaged families, as the physically demanding jobs as well as long and non-standard working hours usually create additional hurdles for fathers to be involved in parenting. With the lack of father–child communication as well as the perception that fathers are less expressive to show their love and affection, father–adolescent discrepancy in perceptions of parental responsiveness is found greater than those of mother–adolescent discrepancies. On the contrary, fathers adopting a goal-oriented style would spell out clear goals and expectations to adolescents, thus reducing father–adolescent discrepancy in parental demandingness.

In contrast to the Chinese culture that emphasizes fathers’ role of training and monitoring the children’s behaviors whereas mother’s roles of caring and nurturing their children, it was found that father–adolescent discrepancy in perceived paternal responsiveness and mother–adolescent discrepancy in perceived maternal control adversely influenced adolescent achievement motivation. We should also be cautious that even small mother–adolescent discrepancy in perceived maternal control would significantly reduce adolescent achievement motivation. Regarding father–adolescent discrepancy of perceived paternal responsiveness in influencing adolescent achievement motivation, the involvement of fathers in adolescence may provide some explanations. In adolescence, fathers engage their children in a “peer-like” manner and are more playful with adolescents (joking and teasing), which promote a more egalitarian father–child exchange and help adolescents develop their own sense of identity and interest (Larson and Richards 1994; Shulman and Klein 1993). Though fathers may not express much affective response to adolescents according to the sex-role theory, their instrumental and goal-oriented concerns to adolescents are critical for their children’s development. This is particularly important for adolescents experiencing economic disadvantage, when adolescents are filially obliged to pursue for excellence as a gratitude to the sacrifice and involvement of their parents (Fuligni and Yoshikawa 2003), especially for their fathers who strive hard for the family. Thus, father–adolescent discrepancy in perceived paternal responsiveness may imply a misunderstanding of adolescents in obtaining paternal support and appropriate concerns, which may induce a loss of achievement motivation of adolescents as a gratitude to their fathers’ involvement.

As far as Chinese indigenous parenting characteristics are concerned, the shame strategy and endorsement of filial piety are distinctive Chinese socialization practices for setting parental expectation and standards in monitoring children’s behaviors under the Chinese culture (Chao 1994; Yang 1981). When parents execute clear standards and rules for adolescents, those who fail to follow would experience shame and guilt (Yang 1981). As a manifestation of filial piety, adolescents may put effort into achievement in order to gain pride and reduce shame to their families (Yu 1996). Since mothers spend more time and effort nurturing their children than do fathers, adolescents would be more sensitive to the standards and rules of mothers. Thus, maternal control has been identified to be a significant predictor of adolescent achievement motivation in Chinese families experiencing economic disadvantage (Leung and Shek 2013). Mother–adolescent discrepancy in parental control may imply a misunderstanding of family standards and expectations among mothers and adolescents, which may lower the adolescents’ motivation to achieve for the sake of the families. On the contrary, the research findings suggested that father–adolescent discrepancy in perceived paternal control did not predict adolescent achievement motivation. With the diminishing role of fathers and expanding role of mothers in monitoring and controlling child’s behaviors in the Chinese communities in recent years (Shek 2007a, 2008), the influence of father–adolescent discrepancy of perceived paternal control in adolescent achievement motivation becomes non-significant. As there is a severe lack of literature on parent–child discrepancies in perceived parenting characteristics in the Chinese communities, the mechanisms of the influences need to be further researched.

Furthermore, we found that mother–adolescent discrepancies in perceptions of maternal responsiveness negatively predicted adolescent psychological competence. With the emphases of familism as well as interdependent relations among family members (Chan and Lee 1995) in the Chinese culture, adolescents develop the concept of self in the linkage of attachment and relationships with the significant others (Ho 1995). The emphasis of interdependence in the Chinese family system was more salient in mother–child relationships, as Chinese mothers emphasized the relational goals of fostering enduring dyadic relationships and sharing of love and affection (Chao and Tseng 2002). Hence, miscommunication or the threat of detrimental mother–child relationships would have adverse effects on adolescent emotional adjustment and development of self-identity. This helps to explain the negative influence of parent–child discrepancy in maternal responsiveness on the psychological development of adolescents.

There are several theoretical implications of the study. First, in view of severe lack of research on the relationship between parent–child discrepancies in perceived parenting characteristics and adolescent psychosocial development in Chinese families experiencing economic disadvantage, the present study is an important addition to the literature. Second, the findings support the view that “informant discrepancies” are absolutely “more than measurement error” (Achenbach 2011, p. 80) and have important meanings and implications on understanding the antecedents of children’s and adolescents’ behaviors (Achenbach 2011; De Los Reyes 2011). The significance of results indicate the importance of treating parent–child discrepancies of parenting characteristics as legitimate constructs, especially when the studies in this area are severely lagging behind. Third, the study sheds new light on understanding familial influences of adolescent psychosocial development in the context of poverty. Regarding how poverty influences adolescent development via family processes, the “family stress” model which emphasizes the mediation pathways of parental distress and poor parenting behaviors (Conger et al. 1994) is dominant in the academic arena. These findings give us an alternate perspective that parent–child discrepancies in perceived parenting characteristics are significant and negatively predicted adolescent developmental outcomes, and provide more information for us to further study the dyadic parent–child interactions within the poor Chinese families. It is obvious that we have to take into the parent–child dyad in understanding the impact of family on adolescent development in the context of poverty. Fourth, the predictions of father–adolescent discrepancy of perceived paternal responsiveness and mother–adolescent discrepancy of perceived maternal control in adolescent achievement motivation suggest a change of parental roles in monitoring and nurturing their children, which provide important ingredients for the exploration of gender parental roles in Chinese families experiencing economic disadvantage.

The study also brings practical implications to family intervention. First, consistent with the previous research (Paulson and Sputa 1996; De Los Reyes et al. 2010) that parents and adolescents may have different perceptions of parenting characteristics, it is important to take into account of different family members in clinical assessment and treatment. Second, research findings indicated that parent–child discrepancies in the perceptions of parenting negatively influenced achievement motivation and psychological competence of adolescents experiencing economic disadvantage. The parent–adolescent discrepancies may be interpreted as problems in parent–child communication that may result in family conflicts and disorganization (Minuchin 1985) in economically disadvantaged families. As suggested by De Los Reyes (2011), informant discrepancies can be important in both understanding the causes and consequences of child and adolescent psychopathology, and allowing treatments to be more focused and appropriate. Hence, clinical practitioners should be sensitive to the differences in the interpretations of parenting between parents and adolescents, as well as the meanings of the discrepancies. Also, they should pay more attention to facilitation of parent–child communication and resolving parent–child conflicts in helping adolescents and families experiencing economic disadvantage. Particularly, the present study identified that father–adolescent discrepancy of perceived responsiveness and mother–adolescent discrepancy of perceived control negatively influenced adolescent achievement motivation, whereas mother–adolescent discrepancy of perceived responsiveness adversely affected adolescent psychological competence, suggesting that clinical practitioners needs to encourage parents to explicitly show their warmth, encouragement and closeness to the needs of their children, and at the same time help the mothers to exercise clear standards and expectations to the adolescents.

Third, the findings call for the family programmes and intervention strategies that can promote parent–child interactions and mutual understanding among family members in economically disadvantaged families. Family life education, asset-building projects for families, parenting enhancement programs, would be necessary.

Last but not least, it was identified that mother–adolescent discrepancies in perceived maternal control and responsiveness have significant negative impacts on achievement motivation and psychological competence of adolescents respectively. Mothers shoulder the dual burdens of caring and monitoring the children, but at the same time they may be blamed for their children’s behaviors (Caplan and Hall-McCorquodale 1985). The overload of maternal roles may be “physically and psychologically taxing for mothers” (Shek 2008, p. 679). The strains and stresses of performing family roles may affect the psychological well-being of mothers, and may result in more parent–child conflicts. The findings alert clinical practitioners to be sensitive to the psychological and parenting needs of mothers and address their needs responsively.

Although this is the first known scientific study examining the relationships between parent–adolescent discrepancies in parenting characteristics and adolescent psychosocial development of Chinese families experiencing economic disadvantage, there are several limitations of the present study. First, the limitation of purposeful sampling should be recognized. As the participated families were not randomly sampled, generalizability of the findings may be limited. Second, the methodological limitation of inviting economically disadvantaged families with poor parenting practice and negative adolescent behavioral outcomes to participate in research may affect the strength of influence of parent–child discrepancies of perceived parenting characteristics in adolescent psychosocial development. Third, the cross-sectional design in this study has the inherent problem in inferring cause-and-effect relationships due to time order. Hence, a longitudinal research design is recommended in future studies. Fourth, the non-random sampling strategy and cross-sectional design of the study bring forth another limitation that some variables (such as level of family stress, learning disability. etc.) that may also be related to developmental outcomes were unable to be controlled. Fifth, as the assessment of parenting characteristics and adolescent psychosocial development was based on the self-reported questionnaires, it is possible that the relationships identified are due to common method variance. Thus, it would be methodological preferable to use multiple methods in future study. Sixth, as the findings presented in the study were based on economically disadvantaged adolescents in Hong Kong, there is a need to assess the generalizability of the findings in different Chinese communities (e.g., mainland China) and Chinese people living in non-Chinese contexts (e.g., Chinese-Americans). Seventh, it was found that the internal consistency of Maternal Demandingness Subscale and Maternal Responsiveness Subscale reported by mothers were not high (although the alpha values were acceptable). This would increase the measurement errors. Hence, it would be desirable to further examine the psychometric properties of these scales in future. Finally, it is noteworthy that the effect size of the significant relationships between discrepancies and psychosocial development was on the low range. Hence, there is a need to interpret the effect of “discrepancies” on perceived parenting characteristics on adolescent psychosocial development in a cautious manner.

Despite these limitations, the present findings are pioneering and stimulating in view of the paucity of research in studying the relationships between parent–adolescent discrepancies in perceived parenting characteristics and adolescent psychosocial development of Chinese families experiencing economic disadvantage. Essentially, the study is an active and constructive response to utilize the parent–child discrepancies in perceived parental behaviors as “legitimate” constructs in exploring their links with adolescent psychosocial development, which sheds light for researchers and clinical practitioners in helping the families experiencing economic disadvantage.
